# Childhood adversities and memory function in later life: the mediating role of activity participation

**DOI:** 10.1186/s12877-024-05145-4

**Published:** 2024-06-21

**Authors:** Xinxin Cai, Xue Bai, Shuai Zhou

**Affiliations:** 1 Department of Applied Social Sciences & Research Centre for Gerontology and Family Studies, The Hong Kong Polytechnic University, Hung Hom, Kowloon, Hong Kong; 2 Department of Applied Social Sciences, The Hong Kong Polytechnic University, Hung Hom, Kowloon, Hong Kong

**Keywords:** Activity participation, Childhood adversity, Cognitive function, Delayed recall, Deprivation and threat, Memory, Older adults

## Abstract

**Background:**

Childhood adversities may lead to decreased activity participation in later life, impacting memory health in ageing adults. Childhood adversities related to deprivation and threat, as conceptualized by the Dimensional Model of Adversity, can exhibit distinct impacts on cognitive and emotional outcomes in children and younger adults. This study examined the potential influence of childhood deprivation and threat on memory function in later life and the mediating role of activity participation in these relationships.

**Methods:**

This study used data from the first wave of Panel Study of Active Ageing and Society (PAAS), a representative survey of Hong Kong residents aged 50 or above (*N* = 1,005). Key variables included late-life memory function measured by delayed recall test, deprivation- and threat-related childhood adversities, and the frequency of participation in informal and formal types of activities. Mediation tests were used for analysis.

**Results:**

Childhood deprivation was associated with a lower late-life memory function, whereas threat was not. The negative effects of childhood deprivation and its subdomain, economic hardship, on memory function were mediated by activity participation. Total participation scores presented the strongest mediating effect (17.3–20.6%), with formal activities playing a more substantial mediating role than informal activities in mitigating the effect of childhood deprivation.

**Conclusions:**

These findings expand the applicability of the Dimensional Model of Adversity to ageing populations, highlighting the influence of deprivation on life-long cognitive development. Furthermore, this study revealed an indirect mechanism by which childhood deprivation affects memory health in old age through diverse activity participation.

**Supplementary Information:**

The online version contains supplementary material available at 10.1186/s12877-024-05145-4.

## Background

Memory function in the ageing population has gained significant attention due to rapid population ageing and increased longevity. This concern is amplified by the prevalence of Alzheimer’s disease, the primary cause of dementia, which has become one of the leading causes of death and disability [1]. Deterioration in memory function can be a natural part of the ageing process, but it can also be a significant early symptom of mild cognitive impairment (MCI) and Alzheimer’s disease (AD) [2, 3]. As a crucial aspect of cognitive function, memory function, particularly episodic memory, is highly sensitive in identifying cognitive deficits [4, 5]. Memory tests, such as the delayed word recall test, have demonstrated superior diagnostic performance in identifying Alzheimer’s Disease, compared to tests targeting other cognitive domains [6–8]. Maintaining sound memory and cognitive health in later life is crucial for healthy ageing, benefiting both individuals and the wider society.

### Childhood adversities and cognitive ageing

Recent literature suggests that adverse early-life conditions and experiences may cast a long-term effect on health and cognition in adulthood because childhood represents a sensitive period in which unfavourable social environments can hinder lifelong neurocognitive development [9]. Three pronged models, encompassing latency, pathway, and cumulative effects, have been developed and widely applied in the investigation of life course influences on health [10–12]. The latency perspective suggests that adverse experiences in early life may exert dormant or delayed effects on health in mid-late adulthood, regardless of life-course interventions [10].

The pathway perspective emphasises the crucial roles of the social, behavioural, and psychological processes that may link childhood adversities to subsequent health outcomes. It posits that early adverse experiences can trigger a chain of risks enduring throughout the lifespan [11], thereby placing individuals on trajectories that impact their long-term health outcomes.

The cumulative disadvantage perspective suggests that the effects of childhood adversities may interact with and be exacerbated by additional life experiences, contributing to a greater risk of negative health outcomes [13]. This means that the negative effect of childhood adversities may compound and accentuate throughout the life course, leading to a cycle where “disadvantages bring more disadvantages” [13, 14], especially when adversities are chronic or co-occurring [15].

In the literature on later-life cognition, the pathway model and cumulative disadvantage perspective have gained increasing traction while evidence for the latency model is rarely reported [16–18]. Childhood adversities have been suggested to lead to subsequent disadvantages in education, relationship formation, and career opportunities throughout life, thereby harming cognitive function in mid to late life [13–15, 20, 21].

Despite a growing body of empirical studies has explored the link between childhood adversities and cognitive function in later life, findings remain mixed [18, 19, 22–28]. The majority of these studies suggest that a range of detrimental experiences, such as poverty, childhood friendships, familial discord, parental absence, and instances of abuse, are predictive of poorer cognitive functioning in old age [19, 24–26]. Some studies suggest that childhood adversities indirectly influence cognition through other factors like education and depression [17, 27], or that the negative effects may not be apparent, potentially compensated by later education and economic status [28]. This evidence, with distinct types of adversities, raises concerns as adverse events can co-occur [29], and the pattern of how cognitive function can be affected remains unclear.

### A dimensional approach to childhood adversities

To comprehensively understand the multifaced nature of childhood adversities and uncover the underlying mechanism of childhood adversities on individual developmental and health outcomes, McLaughlin et al. [30] proposed a *Dimensional Model of Adversity.* This model divides childhood adversities into deprivation and threat. Deprivation refers to the absence or lack of expected environmental inputs (e.g., institutionalisation, neglect, poverty), while threats involve experiences of harm (e.g., abuse, violence, and other traumatic-leading experiences) [29, 31].

Emerging evidence from this dimensional model suggests that deprivation and threat-related adversities affect individuals’ neuro and psychobehavioral development differently. Neurologically, childhood deprivation mainly influences the volume and function of frontoparietal regions, whereas the effects of threat are concentrated around hippocampal, amygdala and medial prefrontal cortex [29, 31]. Consequently, deprivation can hinder cognitive development in individuals who grew up in austerity, affecting their language, executive functioning, and other complex cognitive processes. In contrast, threat can lead to altered functions in detecting and learning about threats or negative emotions, salience processing, and emotional regulation [29, 31]. This dimensional model has been widely discussed and examined [20, 32–35], primarily in children and adolescents. To our knowledge, few previous studies have examined whether deprivation and threat influence cognitive outcomes differently in individuals in their middle to late adulthood.

### Activity participation as a potential mediator between childhood adversities and memory function

While studies have shown that activity participation can help maintain memory and cognitive function [36], individuals who have experienced childhood adversities are often less inclined to engage in activities [26, 37], thereby limiting their opportunities to protect their memory health. This also reflects the cumulative disadvantages of childhood adversities throughout the lifetime, where people with initial exposure to risks can experience sequential adverse conditions later.

The impact of activity participation on cognition is manifested through the enhancement of cognitive reserve. Cognitive reserve refers to the brain’s resilience against damage, which is fortified through education, occupational attainment, and cognitive-stimulated activities [38–41]. Activities such as reading or games serve as direct cognitive and brain exercises, while social activities involving interpersonal communicating require a complex set of cognitive abilities that contribute to cognitive reserve [41]. Sustained engagement in these mentally stimulating activities is beneficial for maintaining an individual’s memory health and preventing age-related cognitive decline [42, 43]. However, individuals exposed to childhood adversities, especially those related to parental and household environments, often face a higher risk of social exclusion in adulthood. This can result in less adaptive personality traits, more behavior problems [44, 45], difficulties in forming and maintaining relationships [46, 47], and weaker social skills and interpersonal trust [47, 48]. Consequently, childhood adversities can lead to smaller and less supportive networks, increased loneliness, reduced activity participation [26, 37, 49], and ultimately poorer cognitive function in older age [26, 50].

While one study has found that participating in diverse activities can mediate the relationship between childhood friendship and episodic memory [26], there is limited evidence regarding the links between childhood adversities, activity participation, and memory function in later life. There is a research gap in understanding how childhood adversities directly influences memory function, while also considering its indirect influence through activity participation. Especially, the relationship between activity participation and cognitive function has yielded conflicting results due to inconsistencies in the inclusion and categorisation of activities. For example, one study found that only mental activities like reading or card games, not social or physical activities, predicted better cognition in older adults [51]. Conversely, another study found that only craft activities help increase cognitive function, not the other 16 types of activities [28]. This highlights the necessity of identifying the specific types of activities that can effectively mitigate the detrimental impact of childhood adversities on memory function.

Previous studies have applied different typologies of activities, such as formal, informal and solitary, or productive and leisure activities [52]. In this study, we categorised activities into two major types: formal and informal activities, based on the mode and purpose of participation. Formal activities refer to organisation-based and productive activities (e.g., activities through association, volunteering), which align with the previously defined formal or serious activities [52, 53]. On the other hand, informal activities involve interactions within informal networks and provide immediate pleasure to individuals on an occasional basis (e.g., interacting with friends or shopping), mirroring previously defined informal or casual activities [52, 53]. No prior studies have examined how childhood adversities differentially influence informal and formal activity participation, despite well-documented evidence that such adversities reduce opportunities for activity participation. To address these limitations, we connected the pathway of deprivation- and threat-related childhood adversities, informal and formal activities, and late-life cognitive function.

### Aims and hypotheses

Using a representative sample of ageing adults aged 50 years or above in Hong Kong, the aim of this study was to examine (a) the influence of deprivation- (i.e., poor health, economic hardship, and parental dysfunction) and threat- (i.e., parental death and addictive behavior, domestic violence, and school bullying) related childhood adversities on late-life memory function; and (b) the mediating role of participation in informal and formal activities in the relationships between childhood adversities and memory function in later life. Identifying the types of adverse events that pose risks for cognitive health in old age and the potential of activities to attenuate the negative influence of childhood adversities, is not only crucial for preventing decline and informing targeted interventions, but also contributes to the understanding of neural changes in brain.

**Fig. 1:**
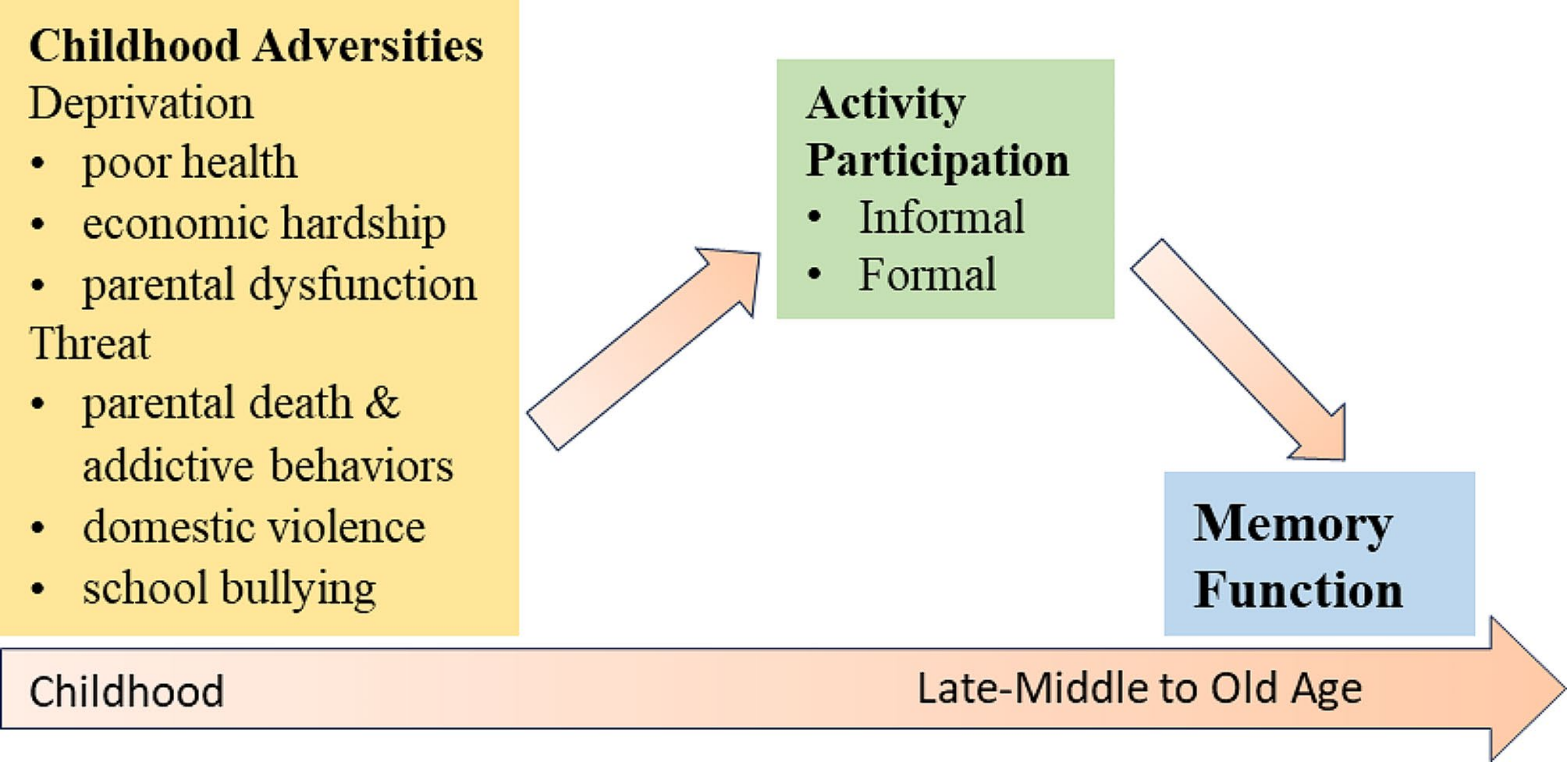
Guiding framework

Figure 1 presents the guiding framework of this study. Based on the three-pronged model of childhood adversities’ impact and the dimensional approach dividing the influence of deprivation and threat, we proposed the following two hypotheses (H):

- ***H1***: *The number of deprivation-related childhood adversities, rather than threat-related adversities, is associated with poorer memory function in later life.*
- ***H2***: *The association between childhood deprivation (and its subdomains) and late-life memory function is mediated by activity participation (both formal and informal activity participation).*

H1 was based on the more prominent influence of deprivation on cognitive function, as indicated by the Dimensional Model of Adversity, aiming to evaluate how early exposure to multiple adversities may cumulatively and differentially affect memory function. With reference to the pathway model, H2 was proposed to investigate the role of activity participation as an indirect pathway through which the number of childhood adversities might affect memory function in later life, and a cumulative risk factor for cognition triggered by childhood adversities.

## Methods

### Study design and participants

Data for this study were drawn from the first wave of ‘Panel Study of Active Ageing and Society in Hong Kong’ (PAAS), which is designed to be a biennial longitudinal survey of ageing adults in Hong Kong [54]. Twenty-six thousand landline and mobile phone numbers were randomly generated and dialed according to the numbering plan of the Office of the Communications Authority, Hong Kong SAR Government. To include Cantonese-speaking adults aged 50 and above, who are cognitively able to answer the telephone survey, the “next birthday” rule was used to select an eligible participant in each household when contact was successfully made. The respondents were equally stratified by two age groups in 50–64 and 65 and over, and the proportion of participants in each age-sex-district stratum was determined based on the Population Census data in 2021. PAAS completed its first wave of data collection between June and November 2022, reaching a total of 8,303 valid phone numbers. A total of 5,007 older adults were successfully interviewed (response rate = 60.3%) by trained researchers. The 3,296 excluded cases included non-contacts (after at least five attempted calls) and refusals. All study procedures and protocols of this non-clinical study involving human subjects were approved by the institutional review board of the first author’s affiliated University (Reference Number: HSEARS20220517001). In this study, we focused on the *N* = 1,005 respondents who participated in the delayed recall test.

### Measures

*Memory Function*. Memory function was assessed using a free delayed word recall test, derived from the Cantonese validated and telephone-administered version of MoCA 5-min protocol [55]. Respondents were asked to memorise a set of five words and recall them after a filled delay of 2 to 3 min. Initially, respondents were asked to freely recall the five words without any cues, with each successful recalled word earning one point. If respondents were unable to recall some or all of the words, they were provided with a semantic cue for the unretrieved words. If this was unsuccessful, multiple-choice hints were given. The final successful recall of words, whether cued or uncued, earned one point. The total score was the sum of free recall and final recall scores, ranging from 0 to 10.

*Deprivation & Threat-related Childhood adversities*. Childhood adversities were assessed using 14 items from the China Health and Retirement Longitudinal Study [56, 57]. Based on the conceptual framework proposed by McLaughlin et al. [29], these adversities were divided into deprivation and threat-related adversities. The deprivation-related adversities consisted of seven items across three subdomains: poor health (including poor health condition and severe disease), economic hardship (such as experiencing starvation and relative economic disadvantage in the community), and parental dysfunction (including parental divorce, mental health issues, and severe disabilities). Conversely, threat-related adversities encompassed seven items categorised into three subdomains: parental death and addictive behaviors (including experiencing parental death in childhood and parental drinking), domestic violence (including physical and mental abuse from parents), and school bullying. Each item was coded as a binary variable (0 = no, 1 = yes). The scores for deprivation-related and threat-related childhood adversities and their respective subdomains were calculated as the sum of the corresponding items, serving as the independent variables in the study. The scoring ranges for deprivation and threat were from 0 to 7.

*Activity Participation*. Activity participation was assessed using respondents’ self-reported frequency of participating in 10 different kinds of activities on a four-point scale, from 1 to 4 (almost every day). These activities were further divided into two types supported by exploratory factor analysis: formal activities (e.g., volunteering, training, social association activities, religious activities, and community-organised activities) and informal activities (e.g., socializing with relatives and friends, playing board games, exercising, yum cha and shopping). The Kaiser-Meyer-Olkin (KMO) value of 0.785 and the significance value obtained from Bartlett’s test of 0.000 indicated that the data was suitable for exploratory factor analysis. The average scores of the respective component items were used. Cronbach’s Alpha for total, informal and formal activity participation was 0.73, 0.69, and 0.75, respectively.

*Covariates*. To account for the potential influences on the relationship between childhood adversities and cognitive function, the study adjusted for factors including age, gender (0 = male, 1 = female), marital status (0 = not married, 1 = married), living with children (0 = no, 1 = yes), retirement status (0 = unretired or others, 1 = retired), estimated years of education, and estimated household monthly income (estimated amount based on the midpoint of the selected income range), self-rated health (on a 5-point Likert scale, 1 = very poor, 5 = very good), loneliness (assessed by the validated Chinese version of the UCLA 3-item loneliness scale [58, 59], and depression (assessed by the validated Chinese version of the CESD-8, the eight-item Center for Epidemiologic Studies Depression Scale [60–62].

### Analysis

Data analyses were performed using SPSS Version 26. Initially, descriptive statistics of the study variables were computed. To represent the population composition of the area, we reported both unweighted and weighted statistics. The weighting was determined using the age, sex, and district population distribution data from the 2021 Population Census. Bivariate tests were then performed to examine the relationships between the key variables. To test the hypotheses, mediation models were analysed using the PROCESS macro of SPSS (Model 4) [63]. First, the mediation role of the total activity participation score was examined in the relationship between deprivation/threat and late-life memory function. For the adversity domain that showed significant indirect effects, further investigation was conducted to explore the mediating role of informal and formal participation, as well as the subdomains of adversities. All mediation models were adjusted for above-mentioned covariates. The coefficients of each path in the models were reported. The level of statistical significance was set at a threshold of *P* < 0.05. To ensure the robustness of the mediation results, sensitivity analyses was performed using KHB decomposition mediation analysis [64] and alternative regression techniques.

## Results

Table 1 presents the sample characteristics. The unweighted mean age of the respondents who participated in the Montreal Cognitive Assessment 5-Minute Protocol Hong Kong Version were 63.37 years old (SD = 7.55), and half of them were women (49.5%; *n* = 497). More than three-fourths of the participants were married (76.6%; *n* = 770) and about half of them were living with children (53.03%; *n* = 533). About 41% of participants have retired (40.7%, *n* = 409). Their average years of education were 9.27 years (SD = 3.23), which denoted a primary middle school level of education. Their average household monthly income was estimated US$3,583.08 (SD = 2,366.94). Their average self-rated health was 3.25 (SD = 0.80) and the mean of loneliness score was 4.30 (SD = 1.58; range 3–9). The average score of depressive symptoms was 5.34 (SD = 3.69).

**Table 1:** Descriptive analysis on the variables included in this study (*N* = 1,005)

	Mean (SD)	
	Unweighted	Weighted
Age	63.37 (7.55)	62.66 (8.10)
Female Gender (0–1)	0.49 (0.50)	0.47 (0.50)
Married (0–1)	0.77 (0.42)	0.77 (0.42)
Living with children (0–1)	0.53 (0.50)	0.56 (0.50)
Retired (0–1)	0.41 (0.49)	0.37 (0.48)
Estimated years of education	9.27 (3.23)	9.39 (3.24)
Estimated household monthly income		
HKD	27966.67 (18474.43)	29300.68 (18617.99)
USD	3583.08 (2366.94)	3753.99 (2385.33)
Self-rated health (1–5)	3.25 (0.80)	3.26 (0.82)
Loneliness (3–9)	4.30 (1.58)	4.24 (1.54)
Depression (CESD-8; 0–24)	5.34 (3.69)	5.31 (3.73)
Memory function (Delayed Recall; 0–10)	7.86 (2.02)	7.90 (2.00)
Childhood adversities		
Deprivation related (0–7)	0.73 (0.88)	0.7 (0.85)
poor health (0–2)	0.10 (0.33)	0.09 (0.32)
economic hardship (0–2)	0.59 (0.68)	0.57 (0.67)
parental dysfunction (0–3)	0.04 (0.24)	0.04 (0.22)
Threat related (0–7)	0.71 (1.17)	0.71 (1.18)
parental death and addictive behaviors (0–2)	0.14 (0.38)	0.14 (0.37)
domestic violence (0–4)	0.52 (0.92)	0.52 (0.93)
school bullying (0–1)	0.05 (0.22)	0.05 (0.22)
Activity participation (1–4)	1.80 (0.37)	1.79 (0.37)
Informal activities (1–4)	2.34 (0.55)	2.32 (0.55)
Formal activities (1–4)	1.25 (0.39)	1.26 (0.39)

Note: SD = standard deviation. The weighting was based on the age, sex, and population proportions of each district derived from the Population Census data in 2021. $1 USD = $7.8052 HKD on 30 November 2022

The mean score of cognitive function assessed by the delayed word recall test was 7.86 (SD = 2.02) out of 10. The average scores for childhood adversities were 0.73 (SD = 0.88) for deprivation-related and 0.71 (SD = 1.17) for threat-related adversities. The mediator, activity participation, had an average score of 1.80 (SD = 0.37). Formal activities were less frequently participated in (mean = 1.25, SD = 0.39) compared with informal leisure activities (mean = 2.34, SD = 0.55).

Table 2 presents the bivariate correlations of the key study variables. Memory function in later life was negatively correlated to childhood deprivation and positively associated with activity participation. Childhood deprivation demonstrated weak but significant positive correlation with activity participation, while threat presented weaker but mixed correlations with formal and informal activities.

**Table 2:** Bivariate test between study variables (*N* = 1,005)

Variables	1. Memory function	Childhood Adversities		Activity Participation		
		2. Deprivation	3. Threat	4. Total	5. Informal	6.Formal
1		-0.20***	0.03	0.21***	0.14***	0.22***
2	-0.19***		0.18***	-0.21***	-0.17***	-0.17***
3	0.01	0.30***		-0.09***	-0.14***	0.04
4	0.21***	-0.20***	-0.03		0.88***	0.67***
5	0.14***	-0.17***	-0.10***	0.87***		0.30***
6	0.22***	-0.15***	0.07**	0.71***	0.27***	

Note: Pearson’s (lower-right) and Spearman’s (upper-left) correlation coefficients are reported. *** *p* < 0.001, ** *p* < 0.01

**Fig. 2:**
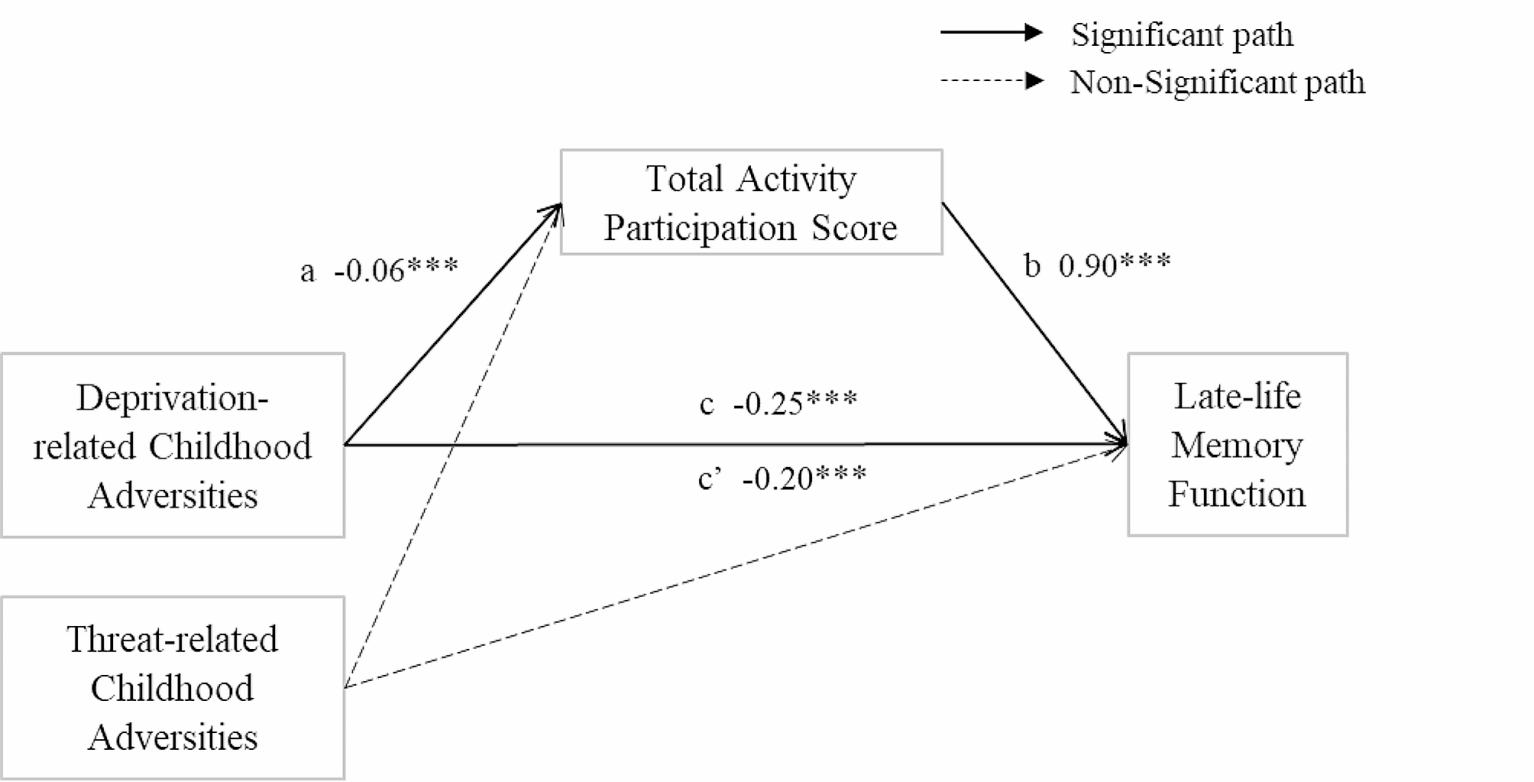
Mediation of childhood adversities and the late-life memory function through activity participation total score (*N* = 1,005) Note: Regression coefficients for significant mediation models reported (the deprivation model). *** *p* < 0.001 (two-tailed tests)

Mediation models were used to examine the role of overall activity participation in the association between childhood adversities and memory function (Fig. 2). Deprivation-related childhood adversities were significantly associated with lower scores in late-life memory function, while threat was not (path c). Deprivation was also found to be associated with lower scores in activity participation, while threat did not (path a). Activity scores were significantly and positively associated with memory function scores (path b). As such, participation in diverse activities partially mediated the relationship between deprivation and late-life memory function, removing 20.6% of the total effect of deprivation. However, no mediation relationship was found for the threat domain. Deprivation-related childhood adversities influence late-life memory function both directly and indirectly through their influence on activity participation.

**Fig. 3:**
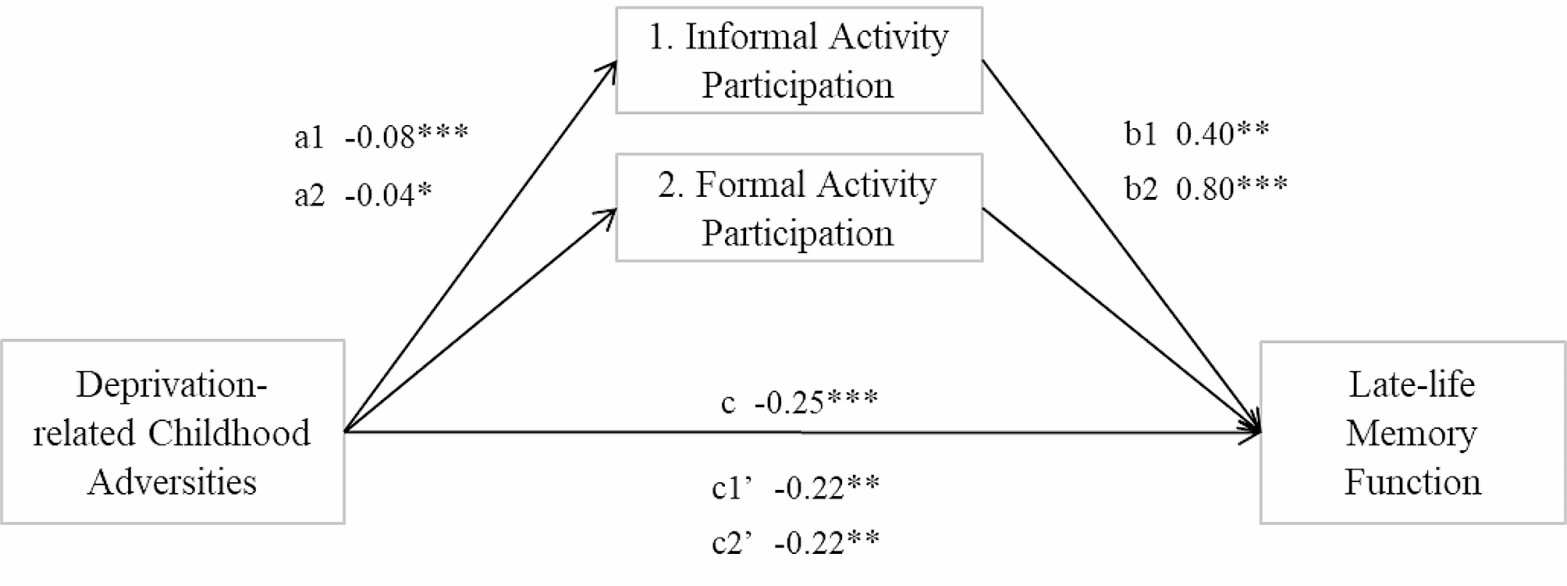
Mediation of deprivation-related childhood adversities and the late-life memory function through subtypes of activity participation (*N* = 1,005) Note: Regression coefficients for significant mediation models reported. *** *p* < 0.001, ** *p* < 0.01, * *p* < 0.05 (two-tailed tests)

We further examined whether the negative influence of deprivation on memory function could be mitigated by different types of activity participation, as depicted in Fig. 3. The findings show that both informal and formal types of activity participation can partially mediate the relationship between deprivation and late-life memory function. The mediating effect accounted for a reduction of 12.4% and 11.7% of the total effect for informal and formal activities, respectively.

**Fig. 4:**
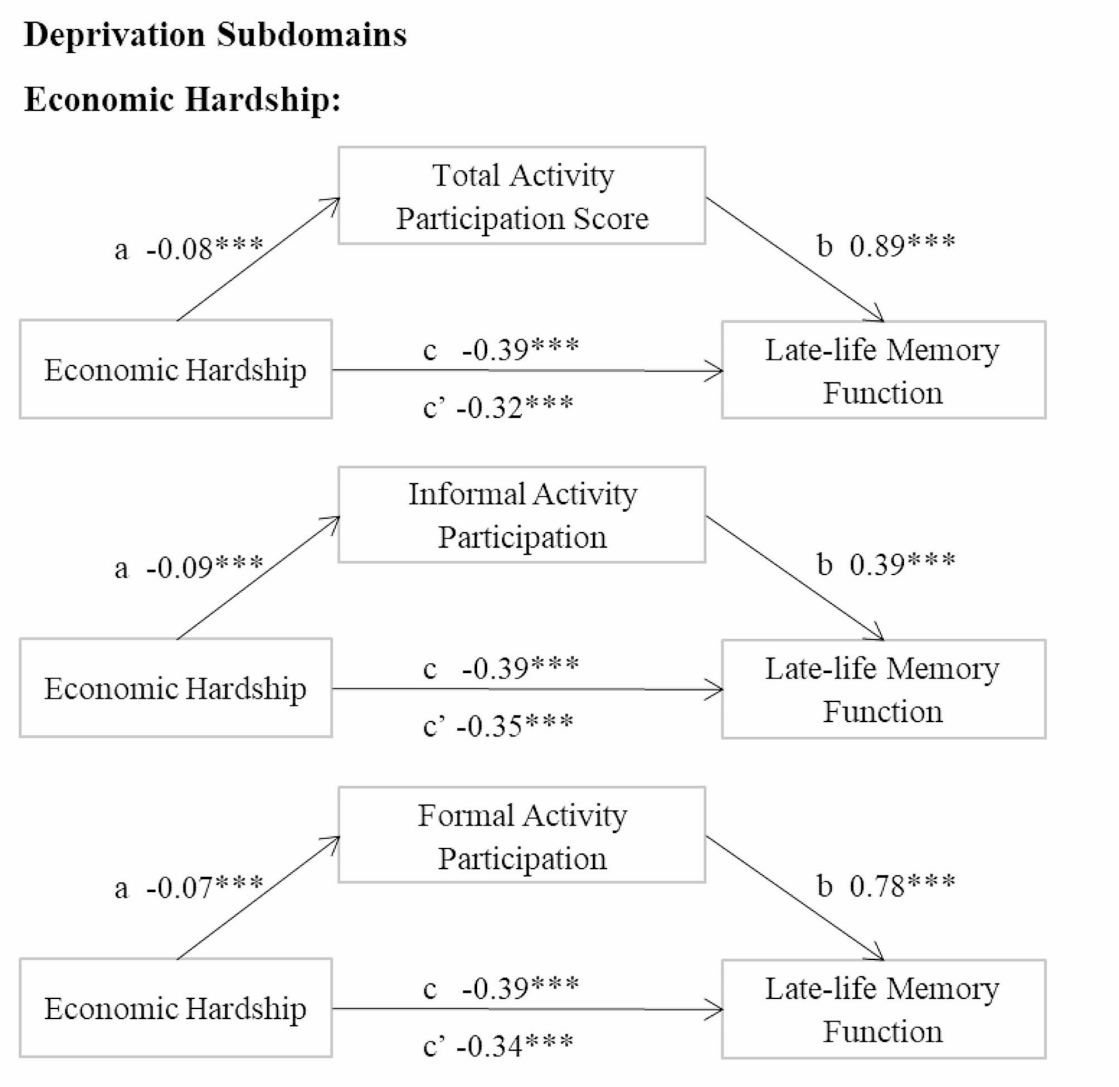
Significant mediating effects of activity participation on the relationship of childhood adversities subdomains and late-life memory function (*N* = 1,005) Note: Coefficients for significant mediation models reported. *** *p* < 0.001 (two-tailed tests)

Additionally, we examined the mediation role of activity participation in the relationship between deprivation and threat subdomains and memory function, as depicted in Fig. 4. For deprivation subdomains, the total effect of economic hardship on memory function was found to be partially mediated by the total activity score, and both informal and formal activities, by 17.3%, 8.7%, and 13.1%, respectively. However, the subdomains of threat-related childhood adversities were not linked to late-life memory function, hence no mediation relationship was observed.

In summary, we found that deprivation, but not threat, was associated with cognitive function in old age, thus supporting Hypothesis 1. Deprivation showed a negative relationship with activity participation, while no significant link was observed for threat. Activity participation was found positively related to memory function. As for mediations, our results suggest that participation in diverse activities, as well as informal and formal activities, can mediate the negative influences of deprivation and its subdomain, economic hardship, on memory function, supporting Hypothesis 2.

### Sensitivity analysis

We first employed KHB decomposition mediation approach to assess the mediation effects of childhood adversities on memory function via activity participation (see Supplementary Table 1). By decomposing indirect effect from the total effect, the KHB method is less biased compared to other mediation analysis methods, and allows the simultaneously examining of more than one mediator [64]. The results of decomposition indicated that total activity participation explained around 20.7% of the associations between deprivation-related adversities on memory function. In the model examining the mediating effects of informal and formal activity participation, it was found that formal activity participation accounted for a slightly larger percent of indirect effect than informal activity participation (10.5% vs. 9.2%). A closer look into key indicators of childhood adversities revealed that economic hardship can affect memory function directly and indirectly through activity participation, particularly formal participation (Supplementary Table 2). In addition, we re-estimated the associations between childhood adversities and respondents’ memory function using nonlinear regression. Poisson models were fitted for the number of unrecalled words and the results showed that only deprivation-related adversities were associated with memory function (Supplementary Table 3). The sensitivity checks suggest that the link between deprivation-related adversities and memory function are robust and can be partly explained by activity participation, especially formal participation.

## Discussion

Using a representative sample of ageing adults in Hong Kong, this study examined the links between threat- and deprivation-related childhood adversities, participation in informal and formal activities, and memory function in later life. The study uniquely applies the categorisation of the Dimensional Model of Adversity to an ageing population, revealing the differential implications of deprivation- and threat-related childhood adversities for older adults’ health outcomes. The results indicated that only childhood deprivation was associated with lower memory function, whereas the influence of threat was not found. Activity participation mediated the relationship between deprivation and late-life memory function, and the effect of its subdomain, economic hardship, could also be mediated by specific types of activities. The most substantial mediating effect was observed in the total activity score, with formal activities having a stronger effect than informal activities. The findings revealed the lifelong detrimental influence of childhood deprivation on memory function, with activity participation partially mediating this relationship. They also highlighted the more potent mitigating role of diverse activity participation.

Consistent with Hypothesis 1, childhood deprivation, rather than threat, negatively influenced memory function in later life. This finding aligns with previous studies on children and adolescents, which found that exposure to deprivation before age 7 was related to poorer cognition and emotional functioning in adolescence [32, 33]. Deprivation from ages 6 to 14 predicted poorer executive function three years later [35], while threat did not. These differences could be due to their distinct impacts on neurodevelopment. Threat tends to alter emotional processing and control, as well as the attentional system, whereas deprivation primarily affects cognitive development [29, 31]. Deprivation is characterised by a lack of material and economic resources, educational opportunities, psychosocial support, and social connectedness, all of which are crucial for cognitive growth [20, 21, 27, 33, 65]. Our bivariate analysis also indicated that deprivation is related to income and education, a connection absents with threat. Moreover, the influence of threat tends to diminish with age, while the effects of deprivation are more enduring [35]. Our study expands the evidence on the influence of deprivation into old age, suggesting that childhood deprivation can have lifelong lasting effects on cognitive functions.

This study also provided valuable insights into the mediating effect of activity participation, suggesting its role as an indirect pathway to later-life cognitive health, as well as one important cumulative risk factor triggered by greater numbers of childhood adversities and subsequently harming memory health as people grow old. Our results tend to support the pathway and cumulative assumption of adversity, rather than latency model. Childhood adversities, especially deprivation, have been associated with inadequate sociability, as presented by a lack of friends, unsatisfactory social relationships, adult disconnectedness, as well as lower educational attainment and socio-economic status [26, 27]. These factors contribute to a cumulative deficit in cognitive reserve, reducing the likelihood of engaging in activities [66] and harming cognitive function [21]. However, participation in activities provides opportunities for social contact, support, and involvement in cognition-stimulating tasks, thereby building cognitive reserve and reducing other risk factors for cognitive decline, such as depression [67]. Essentially, and in line with a similar study on childhood friendship [26], childhood deprivation places individuals at a disadvantage in terms of acquiring cognitive reserve, with part of its negative impact being mediated through reduced activity participation in later life.

Economic hardship, a subtype of childhood deprivation, was found to be negatively associated with both formal and informal participation in later life. Childhood economic status was found to be crucial for cognitive function in later life [68–70]. Our findings align with intriguing evidence suggesting that financial disadvantage in infancy significantly influences cognitive function until mid-childhood, while other impacts fade sooner [33]. It is possible that compared to other domains, poverty that begins in childhood may shape an individual’s inferior socioeconomic status for many years, thereby maintaining a long-lasting effect into old age. Therefore, it is crucial for service providers to recognise the cognitive risks faced by ageing adults who have experienced childhood economic deprivation and provide opportunities for them to compensate for these risks.

Our findings also revealed that total activity scores, representing participating in diverse activities, exerted the strongest mediating effect. In our sensitivity analysis, for both deprivation and economic hardship, formal activities presented a stronger mediating effect than informal activities. These findings echoed evidence in longitudinal study that participation in diverse activities is the best predictor of a decreased risk of dementia [71]. Additionally, formal activities, which are often organisation-based and imply long-term pursuits such as personal development and societal contributions, may be delivered in a more task-oriented manner, providing stronger stimulation for memory and cognitive function. Especially for individuals who have experienced childhood economic hardship and may still be in a disadvantaged socioeconomic status, formal organisations like social services may actively engage and involve them, creating opportunities for social participation. While comprehensive participation is generally recommended, further research is needed to explore how the structure and delivery of formal and informal activities can be optimized to effectively safeguard the cognitive health of older adults.

In this study, we have not identified significant associations between childhood threat and memory function in later life. Exposure to threat may negatively impact the social information processing and emotional functioning [34]. Individuals who have experienced threats may be more sensitive to others’ emotional changes, particularly negative ones, making interpersonal communication more challenging and stressful for them. This could potentially explain why individuals who have experienced childhood threats may not benefit from the protective role of activities. Furthermore, it is possible that unforeseen life events may have occurred, rendering the included activities ineffective in offsetting the impacts of threat. Examining different types of adversities or activities may reveal other unidentified mechanisms. Future research is warranted to identify psychosocial responses to childhood threats and to explore potential effective interventions that facilitate social inclusion while preserving cognitive functioning.

The evidence conveys important messages to inform policy and service that better support the memory health for both older adults and households with children in deprived situations. Meaningful engagement in activities fosters stable and continuous supportive relationships for individuals who have experienced childhood adversities [72]. For older adults, sustained participation in diverse activities, involving in both formal and informal interactions, can create opportunities for cognitive stimulation, social interaction, and personal fulfillment, and social inclusion, offering greater protection for late-life cognition. Policymakers and service providers need to provide platforms to engage older adults in various activities, so that the negative influence of childhood deprivation could be ameliorated.

On the other hand, considering the long-lasting impact of childhood deprivation, especially economic hardship, policies to support people living in poverty should pay attention to households with young children. Additional financial assistance or support programs could be a potential way to ensure equal education and development opportunities that facilitate cognitive development of children from economically disadvantaged households, which may bring life-long benefits for their health and well-being.

### Limitations

Our study makes a novel contribution by examining (a) the influences of two core dimensions of childhood adversities: deprivation and threat, on memory function among ageing adults; and (b) the mediating role of formal and informal activity participation. However, the study is subject to several limitations. First, memory function only represents one aspect of cognitive function, which encompasses a wide spectrum of mental abilities. These include basic cognitive functions such as attention, memory, and perception, as well as higher-level functions like language, decision-making, and executive control [73]. Although memory tests have proven more sensitive in diagnosing Alzheimer’s Disease, further evidence is needed to understand how deprivation and threat influence other domains and overall cognitive function, to provide a more comprehensive understanding.

Second, given the complexity of human development and life experiences, our model captures only a limited number of predictors of memory functioning in ageing adults. We lack information on activity participation during early and middle life, as well as individual personalities and proactive coping strategies for difficulties stemming from childhood adversities, thereby missing the attributes of agents. It is likely that there exist other indirect pathways, for example, marital and intergenerational relationships have been found to mediate the relationship between childhood adversities and depression in Chinese older adults [74]. Future interdisciplinary research is needed to establish a comprehensive understanding that describes how childhood adversities influence cognitive function and decline in later life.

Third, it is crucial to note again that deprivation and threat, as conceptual domains, are not mutually exclusive and can coexist [29]. Although childhood adversities’ measures in our study were adapted from similar items used in established longitudinal studies on ageing [57], they may not cover all relevant types of adverse events in context.

Lastly, due to the cross-sectional nature of the study, we cannot infer causality between late-life activity participation and cognitive function, as the associations could be affected by recall bias. Future longitudinal results of the PAAS and related datasets may provide more robust causal estimates between associated variables using improved identification strategies.

## Conclusion

Our study revealed unique findings that deprivation-related childhood adversities predict poorer memory function in ageing adults. Moreover, diverse activity participation can partially mediate this relationship, with formal activities presenting a slightly stronger effect than informal activities. These findings underscore the long-term effects of childhood adversities on individuals’ psychosocial and cognitive development, and suggest that future research should build on these insights to further examining the detailed mechanisms from a life-course perspective, and develop interventions promoting cognitive health for vulnerable populations.

### Electronic supplementary material

Below is the link to the electronic supplementary material.

Supplementary Material 1

## Data Availability

The dataset analysed in the current study is not currently available to the public. However, it can be obtained from the team led by the second author upon reasonable request.
