# Supplementary material for: Childhood adversities and memory function in later life: the mediating role of activity participation
Source: BMC Geriatr. 2024 Jun 21;24:536. doi: 10.1186/s12877-024-05145-4 (PMC11191259; doi:10.1186/s12877-024-05145-4)
Supplement: Supplementary file 1 — Supplementary Material 1 [file 12877_2024_5145_MOESM1_ESM.docx]

**Supplementary Materials**

**Supplementary Table 1** KHB decomposition results of direct and indirect effects of childhood adversities via activity participation.

|  | (1) | (2) | (3) | (4) |
| --- | --- | --- | --- | --- |
|  | Deprivation | Threat | Deprivation | Threat |
| Reduced | -0.249*** | 0.076 | -0.249*** | 0.076 |
|  | (0.074) | (0.055) | (0.074) | (0.055) |
| Full | -0.197** | 0.072 | -0.200** | 0.061 |
|  | (0.075) | (0.055) | (0.075) | (0.056) |
| Diff | -0.051*** | 0.003 | -0.049** | 0.015 |
|  | (0.016) | (0.010) | (0.016) | (0.012) |
| **Percent of indirect effect through** |  |  |  |  |
| - Total activity participation score | 20.67**%** | - |  |  |
| - Informal activity participation |  |  | 9.16**%** | - |
| - Formal activity participation |  |  | 10.50**%** | - |
| Note: *** *p*<0.001, ** *p*<0.01. | | | | |

**Supplementary Table 2.** KHB decomposition results of direct and indirect effects of key subdomains of childhood adversities via activity participation.

|  | (1) | (2) |
| --- | --- | --- |
|  | Economic hardship | Economic hardship |
| Reduced | -0.386*** | -0.386*** |
|  | (0.096) | (0.096) |
| Full | -0.319*** | -0.315** |
|  | (0.097) | (0.096) |
| Diff | -0.067*** | -0.071*** |
|  | (0.020) | (0.021) |
| **Percent of indirect effect through** |  |  |
| - Total activity participation score | 17.29% |  |
| - Informal activity participation |  | 6.56% |
| - Formal activity participation |  | 11.75% |
| Note: *** *p*<0.001, ** *p*<0.01. | | |

**Supplementary Table 3.** Poisson regression results of memory difficulty on childhood adversities (*N* = 1,005).

|  | (1) | (2) |
| --- | --- | --- |
|  | Objective memory difficulty | Objective memory difficulty |
|  | Poisson | Poisson |
| Deprivation-related adversities | 0.117*** |  |
|  | (0.023) |  |
| Threat-related adversities |  | -0.032 |
|  |  | (0.019) |
| Control | Yes | Yes |
| Intercept | 0.049 | 0.155 |
|  | (0.302) | (0.302) |
|  |  |  |
| Log likelihood | -2008.788 | -2019.813 |
| Pseudo R2 | 0.041 | 0.035 |

Note: Raw coffiicents are reported. Objective memory difficulty was measued by counting the number of unrecalled words. Control variables included age, sex, living arrangement, marital status, retirement, education, household inome, and self-rated health.

*** *p*<0.001, * *p*<0.05.
